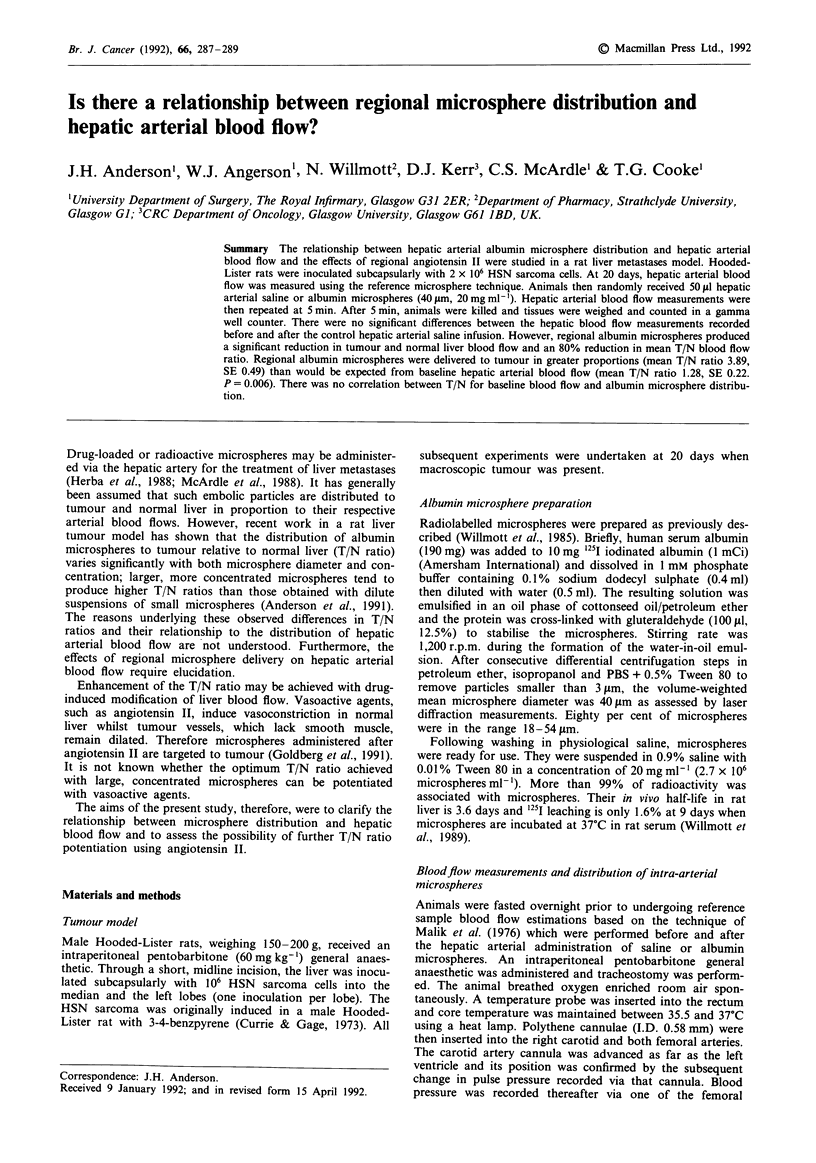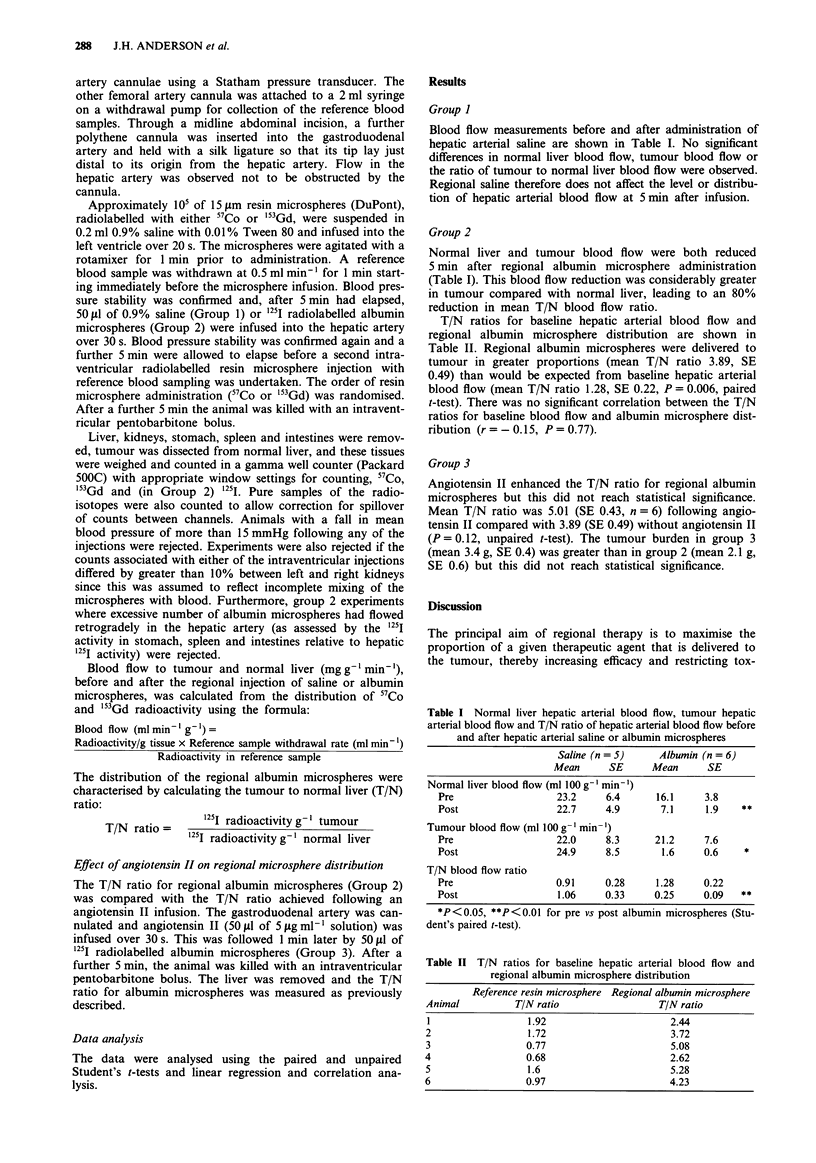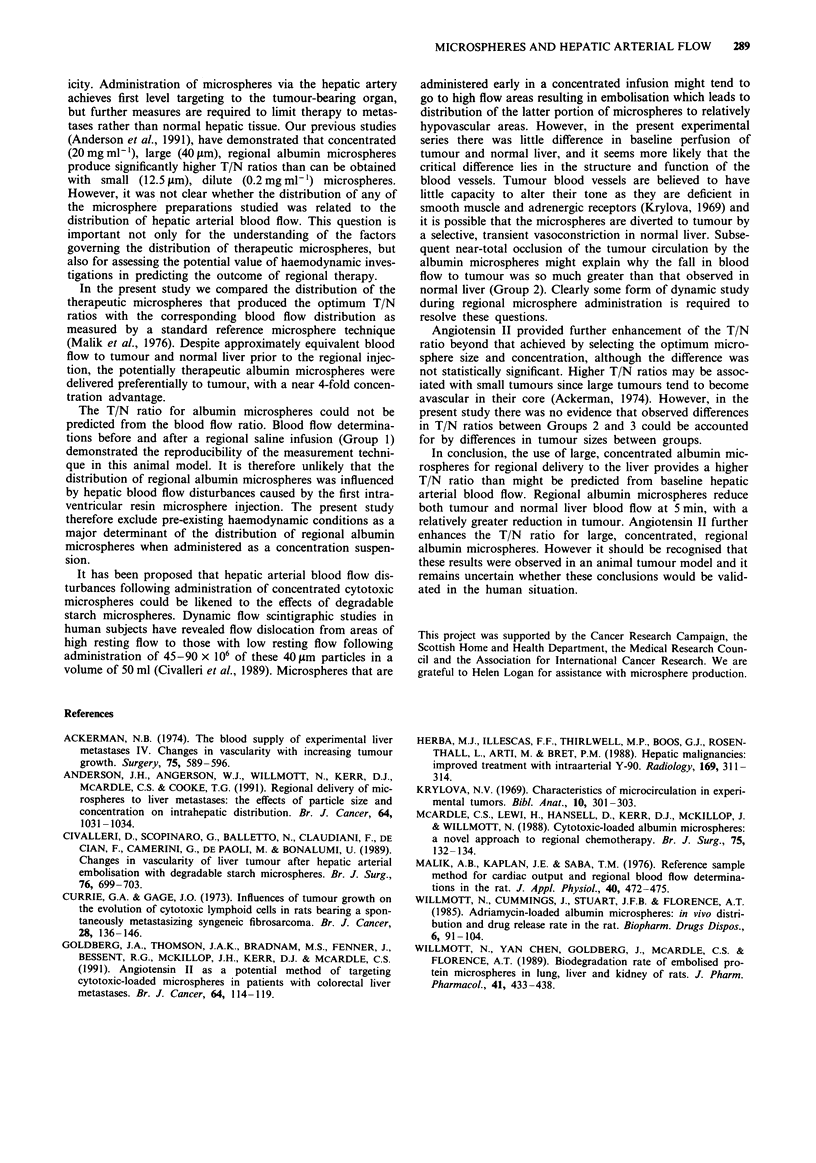# Is there a relationship between regional microsphere distribution and hepatic arterial blood flow?

**DOI:** 10.1038/bjc.1992.258

**Published:** 1992-08

**Authors:** J. H. Anderson, W. J. Angerson, N. Willmott, D. J. Kerr, C. S. McArdle, T. G. Cooke

**Affiliations:** University Department of Surgery, Royal Infirmary, Glasgow, UK.

## Abstract

The relationship between hepatic arterial albumin microsphere distribution and hepatic arterial blood flow and the effects of regional angiotensin II were studied in a rat liver metastases model. Hooded-Lister rats were inoculated subcapsularly with 2 x 10(6) HSN sarcoma cells. At 20 days, hepatic arterial blood flow was measured using the reference microsphere technique. Animals then randomly received 50 microliters hepatic arterial saline or albumin microspheres (40 microns, 20 mg ml-1). Hepatic arterial blood flow measurements were then repeated at 5 min. After 5 min, animals were killed and tissues were weighed and counted in a gamma well counter. There were no significant differences between the hepatic blood flow measurements recorded before and after the control hepatic arterial saline infusion. However, regional albumin microspheres produced a significant reduction in tumour and normal liver blood flow and an 80% reduction in mean T/N blood flow ratio. Regional albumin microspheres were delivered to tumour in greater proportions (mean T/N ratio 3.89, SE 0.49) than would be expected from baseline hepatic arterial blood flow (mean T/N ratio 1.28, SE 0.22. P = 0.006). There was no correlation between T/N for baseline blood flow and albumin microsphere distribution.


Br. J. Cancer (1992), 66, 287-289 © Macmillan Press Ltd., 1992

### Is there a relationship between regional microsphere distribution and hepatic arterial blood flow?

J.H. Anderson[1], W.J. Angerson[1], N. Willmott[2], D.J. Kerr[3], C.S. McArdle[1] & T.G. Cooke[1]

[1]University Department of Surgery, The Royal Infirmary, Glasgow G31 2ER; [2]Department of Pharmacy, Strathclyde University, Glasgow G1; [3]CRC Department of Oncology, Glasgow University, Glasgow G61 1BD, UK.

**Summary** The relationship between hepatic arterial albumin microsphere distribution and hepatic arterial blood flow and the effects of regional angiotensin II were studied in a rat liver metastases model. Hooded-Lister rats were inoculated subcapsularly with $2 \times 10^6$ HSN sarcoma cells. At 20 days, hepatic arterial blood flow was measured using the reference microsphere technique. Animals then randomly received 50 μl hepatic arterial saline or albumin microspheres (40 μm, 20 mg ml$^{-1}$). Hepatic arterial blood flow measurements were then repeated at 5 min. After 5 min, animals were killed and tissues were weighed and counted in a gamma well counter. There were no significant differences between the hepatic blood flow measurements recorded before and after the control hepatic arterial saline infusion. However, regional albumin microspheres produced a significant reduction in tumour and normal liver blood flow and an 80% reduction in mean T/N blood flow ratio. Regional albumin microspheres were delivered to tumour in greater proportions (mean T/N ratio 3.89, SE 0.49) than would be expected from baseline hepatic arterial blood flow (mean T/N ratio 1.28, SE 0.22. $P = 0.006$). There was no correlation between T/N for baseline blood flow and albumin microsphere distribution.

Drug-loaded or radioactive microspheres may be administered via the hepatic artery for the treatment of liver metastases (Herba *et al.*, 1988; McArdle *et al.*, 1988). It has generally been assumed that such embolic particles are distributed to tumour and normal liver in proportion to their respective arterial blood flows. However, recent work in a rat liver tumour model has shown that the distribution of albumin microspheres to tumour relative to normal liver (T/N ratio) varies significantly with both microsphere diameter and concentration; larger, more concentrated microspheres tend to produce higher T/N ratios than those obtained with dilute suspensions of small microspheres (Anderson *et al.*, 1991). The reasons underlying these observed differences in T/N ratios and their relationship to the distribution of hepatic arterial blood flow are not understood. Furthermore, the effects of regional microsphere delivery on hepatic arterial blood flow require elucidation.

Enhancement of the T/N ratio may be achieved with drug-induced modification of liver blood flow. Vasoactive agents, such as angiotensin II, induce vasoconstriction in normal liver whilst tumour vessels, which lack smooth muscle, remain dilated. Therefore microspheres administered after angiotensin II are targeted to tumour (Goldberg *et al.*, 1991). It is not known whether the optimum T/N ratio achieved with large, concentrated microspheres can be potentiated with vasoactive agents.

The aims of the present study, therefore, were to clarify the relationship between microsphere distribution and hepatic blood flow and to assess the possibility of further T/N ratio potentiation using angiotensin II.

### Materials and methods

### Tumour model

Male Hooded-Lister rats, weighing 150–200 g, received an intraperitoneal pentobarbitone (60 mg kg$^{-1}$) general anaesthetic. Through a short, midline incision, the liver was inoculated subcapsularly with $10^6$ HSN sarcoma cells into the median and the left lobes (one inoculation per lobe). The HSN sarcoma was originally induced in a male Hooded-Lister rat with 3-4-benzpyrene (Currie & Gage, 1973). All subsequent experiments were undertaken at 20 days when macroscopic tumour was present.

### Albumin microsphere preparation

Radiolabelled microspheres were prepared as previously described (Willmott *et al.*, 1985). Briefly, human serum albumin (190 mg) was added to 10 mg $^{125}$I iodinated albumin (1 mCi) (Amersham International) and dissolved in 1 mM phosphate buffer containing 0.1% sodium dodecyl sulphate (0.4 ml) then diluted with water (0.5 ml). The resulting solution was emulsified in an oil phase of cottonseed oil/petroleum ether and the protein was cross-linked with gluteraldehyde (100 μl, 12.5%) to stabilise the microspheres. Stirring rate was 1,200 r.p.m. during the formation of the water-in-oil emulsion. After consecutive differential centrifugation steps in petroleum ether, isopropanol and PBS + 0.5% Tween 80 to remove particles smaller than 3 μm, the volume-weighted mean microsphere diameter was 40 μm as assessed by laser diffraction measurements. Eighty per cent of microspheres were in the range 18–54 μm.

Following washing in physiological saline, microspheres were ready for use. They were suspended in 0.9% saline with 0.01% Tween 80 in a concentration of 20 mg ml$^{-1}$ ($2.7 \times 10^6$ microspheres ml$^{-1}$). More than 99% of radioactivity was associated with microspheres. Their *in vivo* half-life in rat liver is 3.6 days and $^{125}$I leaching is only 1.6% at 9 days when microspheres are incubated at 37°C in rat serum (Willmott *et al.*, 1989).

### Blood flow measurements and distribution of intra-arterial microspheres

Animals were fasted overnight prior to undergoing reference sample blood flow estimations based on the technique of Malik *et al.* (1976) which were performed before and after the hepatic arterial administration of saline or albumin microspheres. An intraperitoneal pentobarbitone general anaesthetic was administered and tracheostomy was performed. The animal breathed oxygen enriched room air spontaneously. A temperature probe was inserted into the rectum and core temperature was maintained between 35.5 and 37°C using a heat lamp. Polythene cannulae (I.D. 0.58 mm) were then inserted into the right carotid and both femoral arteries. The carotid artery cannula was advanced as far as the left ventricle and its position was confirmed by the subsequent change in pulse pressure recorded via that cannula. Blood pressure was recorded thereafter via one of the femoral

Correspondence: J.H. Anderson.
Received 9 January 1992; and in revised form 15 April 1992.

artery cannulae using a Statham pressure transducer. The other femoral artery cannula was attached to a 2 ml syringe on a withdrawal pump for collection of the reference blood samples. Through a midline abdominal incision, a further polythene cannula was inserted into the gastroduodenal artery and held with a silk ligature so that its tip lay just distal to its origin from the hepatic artery. Flow in the hepatic artery was observed not to be obstructed by the cannula.

Approximately $10^5$ of 15 $\mu$m resin microspheres (DuPont), radiolabelled with either $^{57}$Co or $^{153}$Gd, were suspended in 0.2 ml 0.9% saline with 0.01% Tween 80 and infused into the left ventricle over 20 s. The microspheres were agitated with a rotamixer for 1 min prior to administration. A reference blood sample was withdrawn at 0.5 ml min$^{-1}$ for 1 min starting immediately before the microsphere infusion. Blood pressure stability was confirmed and, after 5 min had elapsed, 50 $\mu$l of 0.9% saline (Group 1) or $^{125}$I radiolabelled albumin microspheres (Group 2) were infused into the hepatic artery over 30 s. Blood pressure stability was confirmed again and a further 5 min were allowed to elapse before a second intraventricular radiolabelled resin microsphere injection with reference blood sampling was undertaken. The order of resin microsphere administration ($^{57}$Co or $^{153}$Gd) was randomised. After a further 5 min the animal was killed with an intraventricular pentobarbitone bolus.

Liver, kidneys, stomach, spleen and intestines were removed, tumour was dissected from normal liver, and these tissues were weighed and counted in a gamma well counter (Packard 500C) with appropriate window settings for counting, $^{57}$Co, $^{153}$Gd and (in Group 2) $^{125}$I. Pure samples of the radioisotopes were also counted to allow correction for spillover of counts between channels. Animals with a fall in mean blood pressure of more than 15 mmHg following any of the injections were rejected. Experiments were also rejected if the counts associated with either of the intraventricular injections differed by greater than 10% between left and right kidneys since this was assumed to reflect incomplete mixing of the microspheres with blood. Furthermore, group 2 experiments where excessive number of albumin microspheres had flowed retrogradely in the hepatic artery (as assessed by the $^{125}$I activity in stomach, spleen and intestines relative to hepatic $^{125}$I activity) were rejected.

Blood flow to tumour and normal liver (mg g$^{-1}$ min$^{-1}$), before and after the regional injection of saline or albumin microspheres, was calculated from the distribution of $^{57}$Co and $^{153}$Gd radioactivity using the formula:

$$\text{Blood flow (ml min}^{-1}\text{ g}^{-1}) = \frac{\text{Radioactivity/g tissue} \times \text{Reference sample withdrawal rate (ml min}^{-1})}{\text{Radioactivity in reference sample}}$$

The distribution of the regional albumin microspheres were characterised by calculating the tumour to normal liver (T/N) ratio:

$$\text{T/N ratio} = \frac{^{125}\text{I radioactivity g}^{-1}\text{ tumour}}{^{125}\text{I radioactivity g}^{-1}\text{ normal liver}}$$

*Effect of angiotensin II on regional microsphere distribution*

The T/N ratio for regional albumin microspheres (Group 2) was compared with the T/N ratio achieved following an angiotensin II infusion. The gastroduodenal artery was cannulated and angiotensin II (50 $\mu$l of 5 $\mu$g ml$^{-1}$ solution) was infused over 30 s. This was followed 1 min later by 50 $\mu$l of $^{125}$I radiolabelled albumin microspheres (Group 3). After a further 5 min, the animal was killed with an intraventricular pentobarbitone bolus. The liver was removed and the T/N ratio for albumin microspheres was measured as previously described.

*Data analysis*

The data were analysed using the paired and unpaired Student's $t$-tests and linear regression and correlation analysis.

### Results

*Group 1*

Blood flow measurements before and after administration of hepatic arterial saline are shown in Table I. No significant differences in normal liver blood flow, tumour blood flow or the ratio of tumour to normal liver blood flow were observed. Regional saline therefore does not affect the level or distribution of hepatic arterial blood flow at 5 min after infusion.

*Group 2*

Normal liver and tumour blood flow were both reduced 5 min after regional albumin microsphere administration (Table I). This blood flow reduction was considerably greater in tumour compared with normal liver, leading to an 80% reduction in mean T/N blood flow ratio.

T/N ratios for baseline hepatic arterial blood flow and regional albumin microsphere distribution are shown in Table II. Regional albumin microspheres were delivered to tumour in greater proportions (mean T/N ratio 3.89, SE 0.49) than would be expected from baseline hepatic arterial blood flow (mean T/N ratio 1.28, SE 0.22, $P = 0.006$, paired $t$-test). There was no significant correlation between the T/N ratios for baseline blood flow and albumin microsphere distribution ($r = -0.15$, $P = 0.77$).

*Group 3*

Angiotensin II enhanced the T/N ratio for regional albumin microspheres but this did not reach statistical significance. Mean T/N ratio was 5.01 (SE 0.43, $n = 6$) following angiotensin II compared with 3.89 (SE 0.49) without angiotensin II ($P = 0.12$, unpaired $t$-test). The tumour burden in group 3 (mean 3.4 g, SE 0.4) was greater than in group 2 (mean 2.1 g, SE 0.6) but this did not reach statistical significance.

### Discussion

The principal aim of regional therapy is to maximise the proportion of a given therapeutic agent that is delivered to the tumour, thereby increasing efficacy and restricting tox-

**Table I** Normal liver hepatic arterial blood flow, tumour hepatic arterial blood flow and T/N ratio of hepatic arterial blood flow before and after hepatic arterial saline or albumin microspheres

| | *Saline (n = 5)* | | *Albumin (n = 6)* | | |
|---|---|---|---|---|---|
| | *Mean* | *SE* | *Mean* | *SE* | |
| Normal liver blood flow (ml 100 g$^{-1}$ min$^{-1}$) | | | | | |
| Pre | 23.2 | 6.4 | 16.1 | 3.8 | |
| Post | 22.7 | 4.9 | 7.1 | 1.9 | ** |
| Tumour blood flow (ml 100 g$^{-1}$ min$^{-1}$) | | | | | |
| Pre | 22.0 | 8.3 | 21.2 | 7.6 | |
| Post | 24.9 | 8.5 | 1.6 | 0.6 | * |
| T/N blood flow ratio | | | | | |
| Pre | 0.91 | 0.28 | 1.28 | 0.22 | |
| Post | 1.06 | 0.33 | 0.25 | 0.09 | ** |

*$P < 0.05$, **$P < 0.01$ for pre *vs* post albumin microspheres (Student's paired $t$-test).

**Table II** T/N ratios for baseline hepatic arterial blood flow and regional albumin microsphere distribution

| *Animal* | *Reference resin microsphere T/N ratio* | *Regional albumin microsphere T/N ratio* |
|---|---|---|
| 1 | 1.92 | 2.44 |
| 2 | 1.72 | 3.72 |
| 3 | 0.77 | 5.08 |
| 4 | 0.68 | 2.62 |
| 5 | 1.6 | 5.28 |
| 6 | 0.97 | 4.23 |

icity. Administration of microspheres via the hepatic artery achieves first level targeting to the tumour-bearing organ, but further measures are required to limit therapy to metastases rather than normal hepatic tissue. Our previous studies (Anderson *et al.*, 1991), have demonstrated that concentrated (20 mg $ml^{-1}$), large (40 μm), regional albumin microspheres produce significantly higher T/N ratios than can be obtained with small (12.5 μm), dilute (0.2 mg $ml^{-1}$) microspheres. However, it was not clear whether the distribution of any of the microsphere preparations studied was related to the distribution of hepatic arterial blood flow. This question is important not only for the understanding of the factors governing the distribution of therapeutic microspheres, but also for assessing the potential value of haemodynamic investigations in predicting the outcome of regional therapy.

In the present study we compared the distribution of the therapeutic microspheres that produced the optimum T/N ratios with the corresponding blood flow distribution as measured by a standard reference microsphere technique (Malik *et al.*, 1976). Despite approximately equivalent blood flow to tumour and normal liver prior to the regional injection, the potentially therapeutic albumin microspheres were delivered preferentially to tumour, with a near 4-fold concentration advantage.

The T/N ratio for albumin microspheres could not be predicted from the blood flow ratio. Blood flow determinations before and after a regional saline infusion (Group 1) demonstrated the reproducibility of the measurement technique in this animal model. It is therefore unlikely that the distribution of regional albumin microspheres was influenced by hepatic blood flow disturbances caused by the first intraventricular resin microsphere injection. The present study therefore exclude pre-existing haemodynamic conditions as a major determinant of the distribution of regional albumin microspheres when administered as a concentration suspension.

It has been proposed that hepatic arterial blood flow disturbances following administration of concentrated cytotoxic microspheres could be likened to the effects of degradable starch microspheres. Dynamic flow scintigraphic studies in human subjects have revealed flow dislocation from areas of high resting flow to those with low resting flow following administration of 45–90 × $10^6$ of these 40 μm particles in a volume of 50 ml (Civalleri *et al.*, 1989). Microspheres that are administered early in a concentrated infusion might tend to go to high flow areas resulting in embolisation which leads to distribution of the latter portion of microspheres to relatively hypovascular areas. However, in the present experimental series there was little difference in baseline perfusion of tumour and normal liver, and it seems more likely that the critical difference lies in the structure and function of the blood vessels. Tumour blood vessels are believed to have little capacity to alter their tone as they are deficient in smooth muscle and adrenergic receptors (Krylova, 1969) and it is possible that the microspheres are diverted to tumour by a selective, transient vasoconstriction in normal liver. Subsequent near-total occlusion of the tumour circulation by the albumin microspheres might explain why the fall in blood flow to tumour was so much greater than that observed in normal liver (Group 2). Clearly some form of dynamic study during regional microsphere administration is required to resolve these questions.

Angiotensin II provided further enhancement of the T/N ratio beyond that achieved by selecting the optimum microsphere size and concentration, although the difference was not statistically significant. Higher T/N ratios may be associated with small tumours since large tumours tend to become avascular in their core (Ackerman, 1974). However, in the present study there was no evidence that observed differences in T/N ratios between Groups 2 and 3 could be accounted for by differences in tumour sizes between groups.

In conclusion, the use of large, concentrated albumin microspheres for regional delivery to the liver provides a higher T/N ratio than might be predicted from baseline hepatic arterial blood flow. Regional albumin microspheres reduce both tumour and normal liver blood flow at 5 min, with a relatively greater reduction in tumour. Angiotensin II further enhances the T/N ratio for large, concentrated, regional albumin microspheres. However it should be recognised that these results were observed in an animal tumour model and it remains uncertain whether these conclusions would be validated in the human situation.

This project was supported by the Cancer Research Campaign, the Scottish Home and Health Department, the Medical Research Council and the Association for International Cancer Research. We are grateful to Helen Logan for assistance with microsphere production.

### References

ACKERMAN, N.B. (1974). The blood supply of experimental liver metastases IV. Changes in vascularity with increasing tumour growth. *Surgery*, **75**, 589–596.

ANDERSON, J.H., ANGERSON, W.J., WILLMOTT, N., KERR, D.J., MCARDLE, C.S. & COOKE, T.G. (1991). Regional delivery of microspheres to liver metastases: the effects of particle size and concentration on intrahepatic distribution. *Br. J. Cancer*, **64**, 1031–1034.

CIVALLERI, D., SCOPINARO, G., BALLETTO, N., CLAUDIANI, F., DE CIAN, F., CAMERINI, G., DE PAOLI, M. & BONALUMI, U. (1989). Changes in vascularity of liver tumour after hepatic arterial embolisation with degradable starch microspheres. *Br. J. Surg.*, **76**, 699–703.

CURRIE, G.A. & GAGE, J.O. (1973). Influences of tumour growth on the evolution of cytotoxic lymphoid cells in rats bearing a spontaneously metastasizing syngeneic fibrosarcoma. *Br. J. Cancer*, **28**, 136–146.

GOLDBERG, J.A., THOMSON, J.A.K., BRADNAM, M.S., FENNER, J., BESSENT, R.G., MCKILLOP, J.H., KERR, D.J. & MCARDLE, C.S. (1991). Angiotensin II as a potential method of targeting cytotoxic-loaded microspheres in patients with colorectal liver metastases. *Br. J. Cancer*, **64**, 114–119.

HERBA, M.J., ILLESCAS, F.F., THIRLWELL, M.P., BOOS, G.J., ROSENTHALL, L., ARTI, M. & BRET, P.M. (1988). Hepatic malignancies: improved treatment with intraarterial Y-90. *Radiology*, **169**, 311–314.

KRYLOVA, N.V. (1969). Characteristics of microcirculation in experimental tumors. *Bibl. Anat.*, **10**, 301–303.

MCARDLE, C.S., LEWI, H., HANSELL, D., KERR, D.J., MCKILLOP, J. & WILLMOTT, N. (1988). Cytotoxic-loaded albumin microspheres: a novel approach to regional chemotherapy. *Br. J. Surg.*, **75**, 132–134.

MALIK, A.B., KAPLAN, J.E. & SABA, T.M. (1976). Reference sample method for cardiac output and regional blood flow determinations in the rat. *J. Appl. Physiol.*, **40**, 472–475.

WILLMOTT, N., CUMMINGS, J., STUART, J.F.B. & FLORENCE, A.T. (1985). Adriamycin-loaded albumin microspheres: *in vivo* distribution and drug release rate in the rat. *Biopharm. Drugs Dispos.*, **6**, 91–104.

WILLMOTT, N., YAN CHEN, GOLDBERG, J., MCARDLE, C.S. & FLORENCE, A.T. (1989). Biodegradation rate of embolised protein microspheres in lung, liver and kidney of rats. *J. Pharm. Pharmacol.*, **41**, 433–438.